# Linking the thermodynamic temperature to an optical frequency: recent advances in Doppler broadening thermometry

**DOI:** 10.1098/rsta.2015.0047

**Published:** 2016-03-28

**Authors:** Livio Gianfrani

**Affiliations:** 1 Dipartimento di Matematica e Fisica, Seconda Università degli studi di Napoli, Caserta, Italy; 2 INRIM, Istituto Nazionale di Ricerca Metrologica, Torino, Italy

**Keywords:** laser absorption spectroscopy, Doppler effect, spectral line shapes, primary gas thermometry, molecular spectra, collisional effects

## Abstract

Laser spectroscopy in the linear regime of radiation–matter interaction is a powerful tool for measuring thermodynamic quantities in a gas at thermodynamic equilibrium. In particular, the Doppler effect can be considered a gift of nature, linking the thermal energy to an optical frequency, namely the line centre frequency of an atomic or molecular spectral line. This is the basis of a relatively new method of primary gas thermometry, known as Doppler broadening thermometry (DBT). This paper reports on the efforts that have been carried out, in the last decade, worldwide, to the end of making DBT competitive with more consolidated and accurate methodologies, such as acoustic gas thermometry and dielectric constant gas thermometry. The main requirements for low-uncertainty DBT, of both theoretical and technical nature, will be discussed, with a special focus on those related to the line shape model and to the frequency scale. A deep comparison among the different molecules that have been selected in successful DBT implementations is also reported. Finally, for the first time, to the best of my knowledge, the influence of refractive index effects is discussed.

## 1. Introduction

Precision spectroscopy in molecular systems is a very active and fascinating research field, in which tests of fundamental physics are increasingly being performed. Owing to the possibilities of measuring optical frequencies with extraordinary precision, advanced laser-based techniques can be implemented to deeply investigate our physical understanding of the Universe. Playing with molecules, it is possible to look for parity violations associated with the weak force, test quantum electrodynamics in chemically bound systems, measure fundamental constants of nature and look for new physics beyond the standard model. One of the most fascinating applications of precision laser spectroscopy consists of measuring the Boltzmann constant from the shape of an atomic or molecular spectral line.

As is well known, the width and the shape of spectral lines, from an absorbing medium in the gas phase at thermodynamic equilibrium, provide a variety of quantitative information on the gas itself, from both the microscopic and macroscopic points of view. In fact, from the accurate analysis of an absorption profile, it is possible to retrieve the strength of a given line, from which the transition dipole moment between two quantum states can be inferred, and the line centre frequency, providing direct information on the involved energy levels. Nowadays, similar data for diatomic and triatomic molecules are of the utmost importance to test the accuracy of new methods and strategies for *ab initio* calculations [1,2]. Furthermore, if the shape of a given line is accurately recorded as a function of the gas pressure, then line broadening and shifting coefficients can be determined. When dealing with molecules of considerable importance for atmospheric and climate physics, accurate values of these parameters are necessary for gas monitoring applications [3]. In fact, if the spectroscopic parameters of a particular line of a given species are known (as a function of the gas temperature), then the absorber gas density can be accurately determined by using laser absorption spectroscopy [4]. This is a possible way to determine the vertical profiles of water, carbon dioxide and other trace constituents of the Earth atmosphere in air- or balloon-borne experiments through punctual (*in situ*) measurements at different altitudes [4].

In the Doppler regime, namely at low gas pressures, the main source of line broadening is the Doppler effect. This latter leads to the Doppler width of a spectral line, which links the thermal energy to an optical frequency, according to the following equation:
1.1
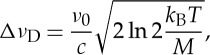
where Δ*ν*_D_ is the Doppler half-width at half maximum (HWHM), *ν*_0_ is the line centre frequency, *c* is the speed of light, *T* is the thermodynamic temperature, *k*_B_ is the Boltzmann constant and *M* is the absorber mass. This equation represents the basis of a relatively new method of primary gas thermometry, known as Doppler broadening thermometry (DBT) [5]. The DBT method consists of retrieving the Doppler width from the highly accurate observation of the shape of a given atomic or molecular line, in a laser-based absorption spectroscopic experiment, under the linear regime of radiation–matter interaction [6]. If the central frequency and the molecular mass are known, by inverting equation (1.1), then it is possible to determine the thermal energy and, consequently, the gas temperature. If implemented at the temperature of the triple point of water (TPW), which is fixed by definition at 273.16 K, the Boltzmann constant can be determined.

In the last 10 years, there has been a growing interest towards the Boltzmann constant, owing to the foreseen redefinition of the unit kelvin, which will be based upon a fixed numerical value for *k*_B_. Before being fixed, *k*_B_ should ideally be determined by several different methods, at a combined uncertainty of 1 part per million (ppm) or smaller. The most accurate way to measure *k*_B_ is from measurements of the speed of sound in a noble gas inside an acoustic resonator [7,8]. After many decades of research and technical developments, acoustic gas thermometry has recently provided a *k*_B_ determination with a relative uncertainty of 0.71 ppm [9]. Another consolidated approach, based upon the Clausius–Mossotti equation, deals with measurements of the electric susceptibility of helium as a function of the gas pressure. Dielectric constant gas thermometry has recently led to a *k*_B_ value with a combined uncertainty of 4.3 ppm [10]. Other primary techniques are presently at the stage of further development and optimization. Among them, DBT is surely the newest one. It should be noted that, in the framework of primary gas thermometry, the development of an optical method linking the thermodynamic temperature to an optical frequency is of the utmost importance to obtain an independent confirmation of the results provided by more established methods. This is the scientific motivation that has stimulated the recent efforts in this field.

Despite the simplicity of the basic idea, the implementation of the DBT methodology is far from being straightforward. Some important requirements of both experimental and theoretical nature should be satisfied for the aims of a successful DBT experiment, as listed here:

(i) the spectral line must be isolated, to avoid line interference effects;
(ii) the detection electronics should be highly linear and sensitive, to ensure sufficiently high fidelity and signal-to-noise ratio in the measured absorption profile;
(iii) the frequency scale underneath each absorption spectrum must be highly reproducible and accurate, to ensure high fidelity (like in the previous item) and to limit as much as possible systematic deviations in Doppler width measurements; and
(iv) the line shape model should be sufficiently refined, so that the Doppler width can be extrapolated with the required accuracy.

The first paper dealing with the application of the DBT technique to the optical determination of the Boltzmann constant was published in 2005, using ammonia (*NH*_3_) as thermometric substance. Probing an isolated line at 10.35 μm, as a result of the analysis of 150 spectra as a function of the gas pressure, *k*_B_ could be determined with an uncertainty of 800 parts over 10^6^, mostly of the type A nature [11]. After 10 years of technical developments, tests and optimizations, accompanied by significant advances in our capability of modelling the spectral line shapes, DBT is nowadays approaching a precision at the ppm level, with solid perspectives of bringing the global uncertainty well below the limit of 10 ppm in the determination of *k*_B_. So far, the best DBT determination of *k*_B_ has been performed on 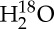 molecules in 2013 and exhibits a global uncertainty of 24 parts per million (ppm), roughly a factor of 30 more accurate, when compared with the first value [12].

In this article, I will report on the current status and new frontiers of research of DBT, also discussing the outcomes of selected experiments. So far, two atomic species and five molecular targets have been tested in DBT experiments. This paper concerns itself with molecules and, primarily, with water and carbon dioxide (two molecules which have been extensively studied in the laboratories of the Second University of Naples), even though many comments will be applicable to DBT in general.

The paper is organized in six sections, besides this introduction and the concluding remarks. The first of them deals with the line shape problem and provides some theoretical arguments to satisfy the fourth of the requirements listed above. The second section is devoted to a wide discussion of three main issues regarding DBT implementations, namely the choice of the molecular target, the wavelength region and the photodetector. Strongly linked one to each other, the three choices can make it possible to satisfy the first two requirements. In the subsequent three sections, the current state of the art in DBT is reviewed, briefly describing recent experiments on carbon dioxide, ammonia and water. In each of them, advanced technical solutions to address the third requirement are highlighted. Finally, in the sixth section, refractive index effects in DBT are discussed for the first time.

## 2. Line shape theory

As is well known, laser absorption spectroscopy consists of the registration of the attenuation of a laser beam travelling into a gaseous medium when tuning the laser frequency in a (semi-) continuous manner over a relatively narrow spectral region around a resonance frequency, corresponding to a transition between two quantum states of a given atomic or molecular species. Near- and mid-infrared absorption spectra of most simple molecules show a large number of highly characteristic rotational–vibrational transitions, which, at sufficiently low vapour pressure and high instrumental resolution, can be easily resolved. The absorption process occurring in the sample cell is ruled by the well-known Beer–Lambert law, which states that the transmitted power *P* decreases as
2.1
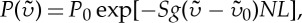
where *P*_0_ is the incident power, 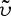 is the laser wavenumber (expressed in cm^−1^), 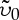 is the line centre wavenumber, *L* is the absorption path length (cm), *N* is the gas density (molecules cm^−3^), *S* is the transition strength (cm per molecule) and 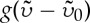 is the line shape function (cm). The latter is normalized, by convention, so that 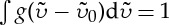. In a gas at thermodynamic equilibrium, the width and the shape of any spectral line is mostly influenced by two main phenomena: the thermal motion of the atomic or molecular constituents and binary collisions among them. Furthermore, depending on the gas pressure, narrowing effects can be observed, typically owing to the joint occurrence of two effects: the speed dependence of collision relaxation rates and the averaging effect of velocity-changing (VC) collisions (the so-called Dicke-narrowing effect).

The most common line shape function is the Voigt function, which is a convolution of the Lorentzian and Gaussian profiles. As such, the Voigt convolution provides a ‘first-order’ representation of both collisional and Doppler broadening, the former under the assumption that the molecules all travel at the mean molecular speed (i.e. neglecting their Maxwellian distribution), and the latter under the assumption that these molecules travel in infinite straight lines (i.e. neglecting collisions). Therefore, pressure and Doppler broadening are treated as statistically independent effects. This first-order representation of the line shape factor produces results that may be satisfactory only when the mean-free path of the absorbers is much larger than the light wavelength, namely in the pure Doppler regime. Nevertheless, depending on the experimental precision in measuring the absorption profiles, deviations from the Voigt model can be observed even in such a regime, as elegantly demonstrated in the case of pure water and pure ammonia samples [13,14]. The ‘second-order’ corrections to these assumptions, for an isolated line under the influence of binary collisions, should consider the occurrence of speed dependence in collisional broadening and shifting, as well as the Dicke-narrowing effect.

Speed dependence of collisional broadening gives a narrowing of the line, whereas speed dependence of collisional shifting leads to line asymmetry. In the formalism developed by Berman and Pickett, collisional parameters are supposed to have a power-law dependence on the relative speed of the absorber/perturber system (*v*_r_), with an exponent determined by the molecular interaction potential, *V* (*r*), which in turn is approximated by an inverse power form [15,16]. More precisely, *V* (*r*)∝1/*r*^q^, with *q*=3, 4 and 5, respectively, for dipole–dipole, dipole–quadrupole and quadrupole–quadrupole interactions, just to cite a few examples; consequently, it turns out that *Γ*(*v*_*r*_)∝*v*^m^_r_ and Δ(*v*_*r*_)∝*v*^n^_r_, *Γ* and Δ being the pressure width and shift, whereas *m* and *n* are given by
2.2
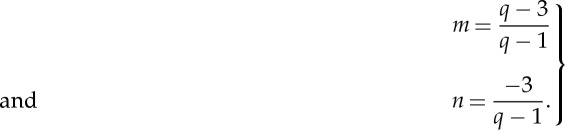
The dependence on the absorbers’ speed can be retrieved by solving the following integral:
2.3
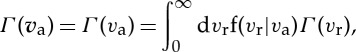
where *f*(*v*_r_|*v*_a_) is the conditional probability distribution of relative speeds. This integral yields
2.4
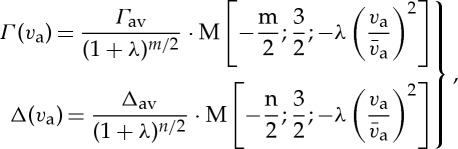
where *λ* is the perturber-to-absorber mass ratio, 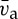 is the most probable speed of the absorbing molecules, *Γ*_av_ and Δ_*av*_ are the average collisional width and shift over the molecular speeds, and M[a; b; z] is the confluent hypergeometric function. Under the impact approximation, in a semiclassical treatment of the spectral line shape, following the pioneering work of Rautian & Sobel’man [17], speed dependence of collisional parameters leads to the so-called speed-dependent Voigt profile (SDVP), given by
2.5
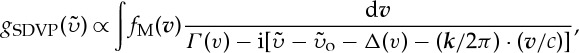
where *f*_M_(***v***) is the Maxwell distribution of the absorber velocities and ***k*** is the wavevector, whose modulus is 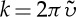.

The physical process of Dicke narrowing, which is due to the averaging effect of VC collisions, can be modelled by using either the soft or the hard collision approximation. In the former case, the effect of an individual collision is infinitesimal, so that several collisions are required to produce a significant change in velocity; consequently, the absorber motion becomes diffusive, thus leading to the Galatry profile [18]. The hard collision limit assumes that each collision completely randomizes the velocity, according to a Maxwellian distribution. This approximation leads to the Nelkin–Ghatak profile [19].

In past literature, it has been frequently shown that ascribing the line narrowing solely to the Dicke effect leads to aberrant values of the VC collision frequency, whose trend as a function of the gas pressure is characterized by unrealistic nonlinearities [20,21]. This is a strong argument to state that the Dicke narrowing alone cannot be sufficient for a physical description of the observed profiles and that speed-dependent effects should be properly accounted for. If one introduces the dipole correlation function, *Φ*(*τ*), which represents the time-domain behaviour of the molecular dipole moment after a pulse excitation occurring at time *τ*=0, the absorption line shape can be seen as the real part of the Fourier transform of *Φ*(*τ*). Ciurylo & Szudy [22] have derived an expression for *Φ*(*τ*), under the soft collision approximation, in order to consider the joint occurrence of the two narrowing mechanisms, thus providing the speed-dependent Galatry profile (SDGP). Similarly, speed dependence can be treated in conjunction with the Dicke effect under the hard collision regime. So doing, the speed-dependent hard collision (or Nelkin–Ghatak) profile (SDHCP) can be obtained [23]. For both the SDGP and SDHCP, VC and dephasing collisions are considered as totally independent. In practice, this is not true: a change of the molecular velocity can be accompanied by a change of the internal state of the collider. This is the case of self-colliding water molecules [24].

In a recent theoretical study, the partially correlated speed-dependent Keilson–Storer (pcSDKS) model has been proposed as a very realistic model to describe *H*_2_O line shapes [25]. Comparisons between simulated spectra and high-quality spectral measurements in the near-infrared (NIR) have shown excellent agreements, thus demonstrating the existence of a partial correlation between VC collisions and rotational-state-changing collisions. This effect is described by a correlation parameter, *η*, which was found to be approximately 0.2 [25]. The complexity and large computational cost of the pcSDKS model do not allow its implementation into a fitting procedure. Instead, it can be used as a benchmark to test the validity of simplified semiclassical profiles. A similar study, described in [26], revealed that the partially correlated speed-dependent hard-collision profile (pcSDHCP) is the most appropriate line shape model for the physical situation of pure H_2_O spectra. Very recently, a simplified version of this profile, in which speed dependence is considered in a quadratic form, has been proposed as a sort of universal line shape model to be adopted for high-resolution spectroscopy in the gas phase [27]. For the sake of simplicity, it was called the Hartmann–Tran profile (HTP) and was judged to be sophisticated enough to capture the various collisional contributions to the isolated line shape.

With the only exception of the HTP, all the cited profiles have been used for the spectral analysis in DBT experiments. It is useful to remember that the observation of non-Voigt effects, especially in the Doppler regime, requires a high signal-to-noise ratio in the spectral recovering, typically larger than 1000 [13], which is also necessary for the aims of low-uncertainty DBT. Despite the recent progress in our capabilities of modelling the profile characterizing an atomic or molecular spectral line, as briefly reviewed in this section, the main source of uncertainty in DBT experiments is still associated with the line shape model, as will be shown later on in the paper.

## 3. Molecules, wavelengths and detectors

As already mentioned, an indispensable prerequisite for a successful DBT experiment is the choice of a well isolated spectral line. To this end, the molecular target has to be as simple as possible, so as to have a restricted number of fundamental modes of vibrations, which normally translates into a simplified structure of its spectrum and a reduced density of spectral lines. Obviously, the selection of the molecular sample is also linked to the choice of the wavelength region. In this respect, the visible and NIR regions should be preferred because of the excellent performance of silicon (Si) and indium–gallium–arsenide (InGaAs) detectors (for radiant powers of less than about 1 mW).

Optical detection is a very critical point for any DBT experiment. Two main features should be carefully considered: the linearity of the response and the noise level at relatively small powers. Si detectors show a linearity over the photocurrent range from 10^−11^ to 10^−3^ A within the expanded uncertainty (corresponding to 2 standard deviations) of 0.054% [28]; similarly, InGaAs photodiodes exhibit a linear response from 10^−7^ to 10^−4^ A within the expanded uncertainty of 0.08% [29]. The linearity was carefully determined by using the flux-addition method with a broadband light source, as explained elsewhere [29]. Depending on their size, even at room temperature, the shunt resistance can easily exceed the limit of 10 MΩ, orders of magnitude larger than that of a germanium (Ge) photodiode, which is another option available for the NIR region [30]. This makes possible the detection of small power levels, namely in the range 1–50 μW, with a sufficiently high signal-to-noise ratio (larger than 1000). It is worth noting that the detection of small light powers requires the selection of a relatively large feedback resistor in the transimpedance amplifier. Differently from InGaAs detectors, such a resistance can overcome the shunt resistance in Ge photodiodes, circumstance that leads to a dominating 1/*f* noise component in the output signal, arising from the operational amplifier [31]. Other important properties of InGaAs detectors are the low dark current (in the nano-ampere range) at room temperature, the high temperature stability and spatial homogeneity, significantly better than those of Ge photodiodes [32]. It is worth to remembering that small incident powers (typically smaller than 50 μW) are required in order to avoid local heating of the gaseous sample that can be a source of systematic deviation in DBT.

The cut-off wavelength of a standard InGaAs detector is typically 1.7 μm (with a lower limit at 0.9 μm); its response can be extended to longer wavelength (up to 2.6 μm at room temperature) by increasing the content of indium [33]. The resulting performance is surely deteriorated, with a dark current density two orders of magnitude larger and a significantly lower shunt resistance (typically smaller than 1 MΩ).

When it comes to the molecular targets, the mid-infrared region, usually referred to as ‘molecular fingerprint’ region, surely offers a number of interesting possibilities. In fact, many molecules exhibit strong and narrow rotation–vibration transitions at wavelengths larger than 3 μm. It is not by chance that the first DBT experiment was performed on ammonia at 10.35 μm. NH_3_ is a pyramidal molecule (symmetry C_3v_) with three identical N−H bonds, showing four fundamental vibrations: the symmetric stretch (*ν*_1_), the symmetric bend (*ν*_2_), the degenerate antisymmetric stretch (*ν*_3_) and the degenerate antisymmetric bend (*ν*_4_). The non-zero spin values of N and H nuclei lead to a hyperfine structure, which must be carefully determined and properly accounted for in the spectral analysis. The symmetric bend, in conjunction with the phenomenon of the inversion doubling, gives rise to two Q branches centred at 950 cm^−1^. Because of the complexity of the molecule, finding a well-isolated spectral line, so as to avoid any deformation of its profile owing to line mixing (or line interference) with neighbouring lines, is not an easy task, even though one can exploit the strong intensities of the rotational components of the fundamental vibrational bands.

The main problem of the mid-infrared region, however, is related to the detectors, which cannot compete with Si or InGaAs photodiodes. The typical detectivity (D*, namely the photosensitivity per unit active area of a detector) of a mercury–cadmium–telluride (MCT) photovoltaic detector (covering the spectral range from 2 to 16 μm and operating at the liquid-nitrogen temperature) is of the order of 3×10^10^ cm (Hz)^1/2^ per W, which is about two orders of magnitude smaller than that of an InGaAs potodiode operating at room temperature. Very recently, the linearity-of-response characteristic of photovoltaic MCT detectors has been the subject of a deep investigation [34]. Depending on the illuminated portion of the active area and on the incident irradiance, deviations from linearity can be as high as 1% (and even larger), in coincidence with a variation of the incident power of approximately 50%. This translates into an instrumental distortion of the line profile and an apparent deviation from the Beer–Lambert law, which has to be properly accounted for in order to avoid systematic deviations in the retrieval of spectroscopic parameters. This is a well-known issue in Fourier-transform infrared spectroscopy, deeply investigated in past literature [35,36].

For a variety of reasons, carbon dioxide is an excellent choice for DBT measurements:

— it is a centrosymmetric and linear molecule (of the 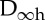 point group) with only three fundamental modes of vibration, thus showing a simplified structure of its infrared spectrum, when compared with other polyatomic molecules;
— there are no hyperfine structure effects; and
— the molecule does not present a permanent dipole moment, circumstance that reduces significantly the interactions with the walls of the gas container.

Apart from the fundamental vibrational bands (*ν*_2_ at 15 μm and *ν*_3_ at 4.26 μm), CO_2_ shows relatively strong absorption lines in the wavelength window between 2 and 3 μm, with typical intensities between 10^−22^ and 10^−19^ cm per molecule. Nevertheless, this spectral region puts some limitations arising from the available detectors, as already discussed above. Higher-order overtone or combination bands can be found at smaller wavelengths, but their intensity decreases by many orders of magnitude, so that their detection in the Doppler regime would require long-path techniques, like cavity-enhanced absorption spectroscopy or cavity ring-down spectroscopy. This is the case of the 
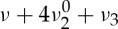
 and 
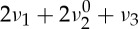
 bands at 1.57 μm.

As a result of this discussion, it seems convenient to look for molecules with absorption features in the spectral interval between 0.9 and 1.7 μm. In this respect, water is surely advantageous because of the relatively small mass, which leads to large vibrational frequencies. As a matter of fact, the spectral lines of the 2*ν*_1_ and *ν*_1_+*ν*_3_ bands occur at 1.4 μm, with an intensity as large as 10^−20^ cm per molecule. Being a light molecule, it also shows a larger Doppler width, when compared with CO_2_ and other heavier molecules with absorption lines in the NIR. Furthermore, the hyperfine structure can be neglected, as magnetic dipole and electric quadrupole effects do not occur. More particularly, hyperfine splitting should be produced for transitions of the ortho type only by spin–spin and spin–rotation interactions [37]. Nevertheless, these effects are so small that the resulting splitting is roughly four orders of magnitude smaller than the Doppler width of the line, whereas the hyperfine structure is totally absent for para transitions [38].

Acetylene is another good candidate, being a non-polar and linear molecule. Despite the relatively large number of vibrational modes (five in total, namely the symmetric C−H stretch, the symmetric C−C stretch, the antisymmetric C−H stretch, the antisymmetric bend and the symmetric bend), it is possible to find well isolated transitions at 1.54 μm, where the molecule presents the widely investigated *ν*_1_+*ν*_3_ band. Rotational components of this band in the 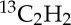 molecule attracted the interest of the International Community for Weights and Measures, which was looking for a primary wavelength standard for the important field of optical telecommunications. In 2001, the Consultative Committee for Length included lasers stabilized to 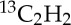 in the list of radiations for the practical realization of the definition of the metre [39]. With such motivation, the line centre frequency of the P(16) component was measured by using nonlinear spectroscopy in conjunction to an optical frequency comb synthesizer, thus reaching a sub-kHz accuracy [40]. The same line was selected by Yamada *et al.* [41,42] for high precision line profile measurements exploiting the technology of erbium-doped fibre-laser frequency comb synthesizers. In their apparatus, an extended-cavity diode laser (ECDL) at 1.5 μm was phase locked to the comb and continuous frequency scans (of about 2 GHz) around the P(16) line were performed by tuning the repetition rate. A precision in Doppler-width retrieval of 1200 ppm was demonstrated by using this approach, mostly limited by the simplified spectral analysis procedure (based upon the Voigt convolution) that was applied to a restricted number of spectra (only 20, in the pressure range between 40 and 650 Pa) [42]. A much simplified experimental set-up was recently implemented by a Canadian group to probe the P(25) line of the C_2_H_2_ *ν*_1_+*ν*_3_ band, in the same wavelength region [43]. The frequency scale underneath the absorption spectra was built by using a Fabry–Perot interferometer in conjunction with a wavelength meter. Despite the inaccuracies in the frequency scale, the global uncertainty in the spectroscopic determination of *k*_B_ was found to be 87 ppm. This rather good determination was possible thanks to the spectral analysis procedure, based upon the use of the speed-dependent Voigt profile, with *q*=5.

Table 1 compares the different molecules mentioned so far. With the only exception of oxygen, all of them have been already tested for the aims of DBT, more or less successfully. Complete details about the spectroscopic parameters of the selected lines are reported. Unless differently indicated, these data are taken from the HITRAN database [44]. Doppler widths and pressure broadening coefficients (*γ*) are reported at the temperature of the TPW, whereas line intensity factors refer to the HITRAN temperature, *T*_0_=296 K. Pressure broadening data have been properly rescaled with the temperature, when compared with the HITRAN value, according the following equation:
3.1
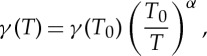
the exponent *α* being 0.7 for linear molecules, 1 for ammonia and 0.75 for water [45].

**Table 1.** Comparison among the different molecules and lines that have been either tested or proposed for DBT experiments.

molecule	line (vibrational and rotational assignments)	frequency (cm^−1^)	pressure broadening coefficient (kHz Pa^−1^)	Doppler width FWHM (MHz)	line strength (cm per molecule) *T*=296 K	permanent dipole moment (C m)	hyperfine structure effects
NH_3_	*ν*_2_ saQ(6,3)	965.79128	264.82	83	6.547×10^−20^	5.053×10^−30^[46]	present
CO_2_	*ν*_1_+2*ν*_2_+*ν*_3_ R(12)	4987.308353	66.35	267	1.222×10^−21^	0	absent
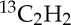	*ν*_1_+*ν*_3_ P(16)	6483.470954 [47]	84.50^a^	435	2.539×10^−21^^a^	0	small
C_2_H_2_	*ν*_1_+3*ν*_3_ R(9)	12 696.4 [48]	80.12 [48]	883	4.493×10^−24^[48]	0	small
C_2_H_2_	*ν*_1_+*ν*_3_ P(25)	6490.020179	68.86	452	1.375×10^−21^	0	small
H_2_^18^O	*ν*_1_+*ν*_3_ 4_4,1_→4_4,0_	7199.103190	210.54	571	6.218×10^−21^	6.184×10^−30^[1]	small
O_2_	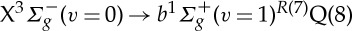	14 546.003919	30.05	913	6.033×10^−25^	0	absent

^a^Spectroscopic parameters of the P(16) line of 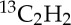 are supposed to be identical to those of the same line of the C_2_H_2_ molecule.

**Figure 1.**
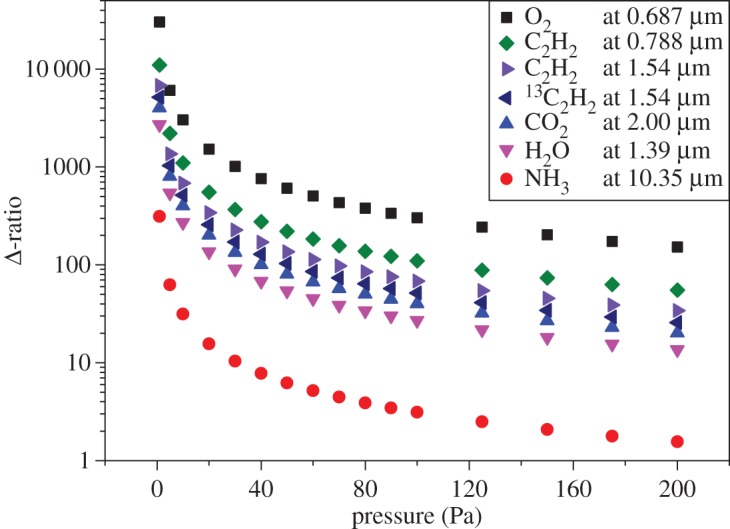
Ratio between Doppler and collisional widths as a function of the gas pressure, for all the molecular lines that have been either used or proposed for a spectroscopic determination of the Boltzmann constant. The higher the ratio is, the less sophisticated the spectral analysis is required to be. The values refer to the spectral lines of table 1. (Online version in colour.)

In figure 1, the ratio between Doppler and collisional widths (here named Δ-ratio), at the TPW temperature, is plotted as a function of the gas pressure, in the interval between 1 and 200 Pa, for the lines and molecules of table 1. The selected pressure range is suitable (and therefore can be explored) in any case. Obviously, the optical path-length is the variable quantity, being smaller (or longer) for the stronger (or weaker) absorbers. More precisely, the path-length can vary from a few tens of centimetres (for NH_3_ in the mid-infrared) at up to a few kilometres (for *C*_2_H_2_ and O_2_ in the NIR and visible regions, respectively), these latter values being attainable by means of high finesse optical cavities, as explained hereafter. A large ratio leads to a simplified line shape model for the aims of line fitting and Doppler-width retrieval. The NH_3_ situation is clearly not advantageous, from this point of view. It is necessary to work at pressures equal to or smaller than 1 Pa in order to get a Δ-ratio larger than 100. This means that speed-dependent effects of collisional broadening should be carefully considered for pressures larger than 1 Pa, as already evidenced in [14]. More favourable ratios can be obtained from water, in the Doppler regime. However, also in this case, for vapour pressures larger than 100 Pa, the Δ-ratio becomes smaller than 30, thus suggesting the necessity of using sophisticated line shape models.

**Figure 2.**
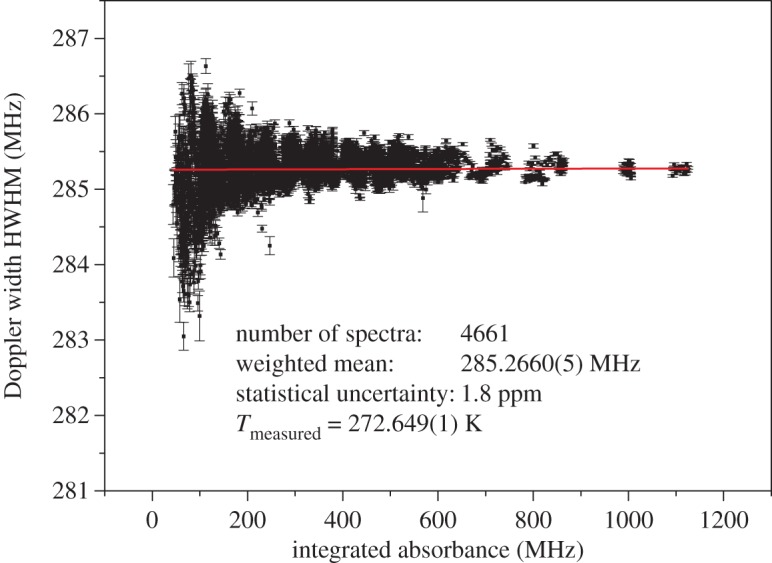
Doppler width determinations from the dual-laser absorption spectrometer at 1.4 μm. The spectral analysis was performed by using the symmetric version of the speed-dependent Voigt profile. Error bars, corresponding to one standard deviation, are the internal errors resulting from the fitting procedure. The relative statistical uncertainty is close to the part per million level, but the spectroscopic measurement of the gas temperature is clearly affected by a negative shift of about 0.5 K. (Online version in colour.)

Figure 2 strongly supports the statement according to which the smaller is the Δ-ratio the more sophisticated is the line shape model that is required. It shows the outcomes of spectral analysis by means of the symmetric version of the speed-dependent Voigt profile performed on 4661 absorption spectra. These data were obtained at the Second University of Naples by using a dual-laser water spectrometer operating at 1.4 μm, described in detail elsewhere [12,49]. The weighted mean of the Doppler widths of figure 2 gives the extraordinary result of 1.8 ppm in terms of statistical uncertainty (calculated as the standard deviation of the weighted mean), but the value itself is affected by a negative shift. This is the clear consequence of the fact that line narrowing effects are not taken into account properly by the simplified model. In fact, using the recommended value for *k*_B_, one would retrieve a spectroscopic temperature of 272.649(1) K, rather than the expected value of 273.16 K.

Going back to figure 1, it is clear that the best Δ-ratios are provided by oxygen at 687 nm, followed by acetylene at 788 nm. Being a homonuclear molecule, O_2_ is not active in the infrared portion of the electromagnetic spectrum. The oxygen molecule has two low-lying excited electronic states, the *a*^1^Δ_*g*_ and 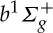, which are approximately 7888 cm^−1^ and 13 122 cm^−1^, respectively, above the 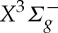 ground state. Weak magnetic dipole transitions are possible between these electronic states, even though those from the ground states (*X*→*a* and *X*→*b*) violate the selection rule on the electronic spin (ΔS=0). As a matter of fact, there are a number of rovibronic transitions in the visible and NIR spectral regions, which are characteristic of the absorption spectrum of solar radiation by the Earth's atmosphere. In particular, the A and B bands (*v*′′=0→*v*′=0 and *v*′′=0→*v*′=1, respectively, of the *X*→*a* system) present many lines that are very well isolated and easily accessible by means of commercially available ECDLs. As reported in table 1, one of them has been proposed for a spectroscopic determination of *k*_B_ [50].

If the O_2_-based implementation is still at the stage of a proposal, there has been already a test experiment on acetylene at 788 nm with a gas pressure ranging from 0.5 to 3 Pa (in which the Δ-ratio is larger than 1000). This experiment yielded spectroscopic measurements of the gas temperature, with the cell operating at room temperature, with a global uncertainty of two parts over 10^3^ [51]. For either O_2_ at 687 nm or C_2_H_2_ at 788 nm, owing to the very small line intensity factors, the use of frequency-stabilized cavity ring-down spectroscopy is necessary. This technique has to be preferred to other cavity-enhanced approaches because it provides a direct (and absolute) measurement of the absorption coefficient, namely the product 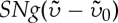 [52]. It is worth noting that the use of an optical cavity requires a particular care. In fact, the enhancement of the absorption path-length is usually accompanied by an enhancement of the optical power inside the cavity [53]; depending on the incident power and mode matching parameter, the intensity can reach extraordinarily high values in the beam waist (easily larger than 10^6^ W m^−2^ for 1 mW of incident power), thus leading to nonlinear effects, on the one hand, and local heating of the absorbing gas, on the other hand. This latter phenomenon, in particular, may lead to a measurable positive shift in the retrieval of the Doppler width.

## 4. The carbon dioxide experiment

**Figure 3.**
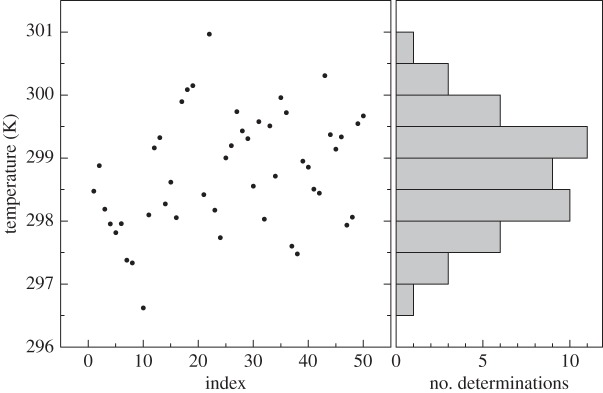
Temperature measurements from CO_2_ absorption spectra at 2 μm wavelength, performed at the Second University of Naples. The mean value is 298.77 K, with a statistical uncertainty (given by the standard error of the mean) of 0.13 K. Spectral analysis was performed by using the Voigt convolution. The precision is far from that of figure 2, but the experimental set-up was much simpler when compared with the dual-laser water spectrometer.

Figure 1 also indicates that the use of *CO*_2_ at 2 μm wavelength (whose lines belong to the *ν*_1_+2*ν*_2_+*ν*_3_ combination band) may be advantageous, the Δ-ratios being larger than those of ammonia and water. In fact, at a pressure of approximately 100 Pa, this ratio amounts to about 40, which, in conjunction with the fact that collisions are much less effective (the molecule being non-polar), explains the success of the experiment that immediately followed the first DBT implementation [54]. In that experiment, the shape of the well isolated R(12) component was probed at different temperatures, between the TPW and the gallium melting point (302.9146 K), by using an ECDL. This spectral line was also selected by researchers at the PTB-Braunschweig for laser-spectroscopy-based measurement of the amount of substance fraction of CO_2_ in N_2_ [55]. Figure 3 shows an example of repeated determinations of the gas temperature, as retrieved at a pressure of about 100 Pa from the analysis of 50 spectra by means of a Voigt profile; the gas cell was temperature stabilized at 298.68(1) K, which was measured by using a standard platinum-resistance thermometer. The histogram on the right side shows that the distribution is Gaussian. The mean value is 298.77 K, with a standard deviation of the mean of 0.13 K, in a satisfactory agreement with the set point [54]. The use of the Voigt profile can be justified by the fact that the mean free-path of the molecules, 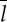, is much larger than *λ*/2*π*. From the kinetic theory of gases, assuming that the molecules behave like hard spheres, it turns out that 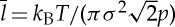, *σ* and *p* being the molecular diameter and the gas pressure, respectively. Using the 12–6 Lennard-Jones intermolecular potential, it is possible to calculate the classical second virial coefficient, from which *σ* can be determined [56]. So doing, the mean free path results to be 51 μm, which is a factor of approximately 160 larger than *λ*/2*π*. A confirmation about the validity of the Voigt model for the aims of Doppler-width retrieval is found in [57], for the same CO_2_ line in the Doppler regime. The CO_2_ experiment of the Second University of Naples provided a spectroscopic determination of the Boltzmann constant with a global uncertainty of 160 ppm, the retrieved value being 1.38058(22) 10^−23^ J K^−1^ [54].

## 5. The ammonia experiments

After the first attempt of 2005 [11], the French group published a very promising result on ammonia, 2 years later [58]. The selected absorption line was the sQ(6,3) component (*J*=6 and *K*=3 being respectively the quantum numbers associated with the rotational angular momentum and its projection on the molecular symmetry axis) of the *ν*_2_ band. This line can be considered a rather good choice, as the neighbouring lines (arising from hot bands) are orders of magnitude weaker. More particularly, among the closest lines, only one of them exhibits a remarkable intensity, its frequency being roughly 2250 MHz away, at higher frequencies, with a line strength of 3.751×10^−24^ cm mol^−1^ (namely more than four orders of magnitude weaker than the probed line). Experimental line profiles were recorded at the temperature *T*=273.15 K, in the pressure interval between 1 and 10 Pa, using a frequency-stabilized CO_2_ laser in conjunction with a microwave electro-optic modulator. This latter generated tunable sidebands (from 8 to 18 GHz) on both sides of the fixed laser frequency, whereas an optical cavity was used as a high-quality spectral filter to get rid of the carrier and of one of the two sidebands. The other sideband is tuned close to resonance with the desired molecular transition and scanned to record the full Doppler profile. The French group provided a determination of the Boltzmann constant with a global uncertainty of 190 parts in 10^6^, resulting from the analysis of 2000 spectra by means of a Gaussian profile [58]. The *k*_B_ value was retrieved from the extrapolated zero-pressure value of the Doppler width, which exhibited an unrealistic linear trend as a function of the gas pressure, clearly owing to the inadequacy of the adopted line shape model. In subsequent papers, in order to overcome this limitation, the spectral analysis was more and more refined, adopting the Voigt convolution [59], the Galatry profile [60] and more recently, the speed-dependent Voigt model [14]. New spectral acquisitions were also performed at much lower pressures (between 0.2 and 2.5 Pa), using an isothermal absorption cell with a multi-pass configuration (thus ensuring an optical path-length of 3.5 m) [61]. In this latter case, the spectral analysis was performed by using the first-order Taylor expansion of the exponential of a Galatry profile, leading to a spectroscopic determination of *k*_B_ with a statistical uncertainty of 6.4 ppm, as obtained from the notable number of 7171 spectra. The best determination, however, was obtained by the French group from spectral measurements (1420, in total) in the pressure interval from 0.1 to 1.3 Pa, the retrieved value being 1.380704(69) 10^−23^ J K^−1^, characterized by a global uncertainty of 50 ppm [61]. Very recently, a revised uncertainty budget has been presented, discussing the main systematic effects and demonstrating the possibility of reaching a combined, type-B, standard uncertainty as small as 2.3 ppm [62]. Nevertheless, this latter paper did not report a new value for *k*_B_. Therefore, the best French determination still remains that of [61].

**Figure 4.**
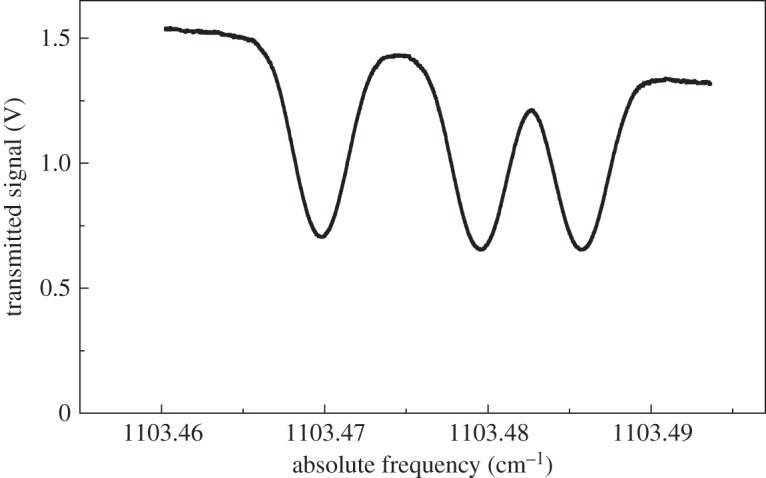
Experimental spectrum obtained by means of comb-assisted QCL-based absorption spectroscopy. The gas cell was 11 cm long. The NH_3_ gas pressure was 13 Pa, whereas the temperature of the cell was 295.18(1) K, as measured by means of a platinum resistance thermometer connected to a 6.5-digit voltmeter.

A highly linear, repeatable and accurate frequency scale represents an indispensable prerequisite for a successful DBT experiment. The French approach ensures high spectral purity of the probe laser (the CO_2_ laser being tightly stabilized against an OsO_4_-saturated absorption line) but it has the drawback of a limited scan width that cannot exceed 250 MHz. A more versatile scheme was implemented by Gatti *et al.* [63] exploiting the technology of thulium optical frequency comb synthesizers. More particularly, a room-temperature continuous-wave distributed feedback quantum cascade laser (QCL) at 9.07 μm was coherently phase locked to the Tm:fibre comb by means of a sum-frequency generation (SFG) scheme. A portion of the femtosecond *Tm*:fibre laser at 1.95 μm is collinearly combined with the QCL and focused by an off-axis parabolic mirror into a AgGaSe_2_ crystal so as to produce a SFG mini-comb at 1.6 μm. The remaining portion of the femtosecond pulse is coupled into a highly nonlinear fibre with zero dispersion wavelength close to 1.95 μm in order to generate supercontinuum (SC) radiation between 1.1 and 2.4 μm. The 1.6 μm radiation is then heterodyned with the SC radiation, after spectral filtering at the wavelength of interest, thus producing a beat-note which is detected by means of a fast photodiode. The phase lock between comb and QCL is achieved by comparing the beat-note signal with a radiofrequency (RF) local oscillator and by feeding the error signal back to the QCL current. So doing, the QCL frequency could be precisely and repeatedly tuned over several GHz by varying the comb repetition rate. Figure 4 shows an example spectrum associated with three closely spaced NH_3_ transitions, namely the sR(6,2), sR(6,6) and sR(6,1) lines of the *ν*_2_ band. Temperature determinations have been demonstrated by using this approach, with a precision of five parts over 10^5^ [64]. The experiment, however, was limited by a negative shift as large as two parts over 10^3^, probably ascribed to the occurrence of line-mixing effects [65].

## 6. The water experiment

The dual-laser water spectrometer consists of an ECDL, namely the probe laser, which is offset-frequency locked to a reference ECDL [49]. This latter is frequency stabilized against the central frequency of a narrow Lamb dip, in coincidence with the 4_4,0_→4_4,1_ line of the 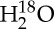 *ν*_1_+*ν*_3_ band at 7198.988700 cm^−1^, following the method described elsewhere [66]. Nonlinear interaction is produced inside a high finesse optical cavity (with an empty-cavity finesse of approx. 5000), after filling it with a 97% ^18^*O*-enriched water sample, at a pressure of approximately 5 Pa.

The absolute stability of the reference oscillator was carefully determined by comparing its emission frequency, *ν*_ref_, to the closest tooth of a self-referenced fibre-based optical frequency comb, whose repetition rate and carrier-envelope offset frequency were stabilized against a 10 MHz GPS-disciplined Rb clock. The Allan deviation analysis demonstrated a relative stability of 10^−12^ for an integration time of 100 s, limited by the stability of the Rb clock. For longer integration times, the observed stability slightly deviated from that of the Rb microwave reference because of the occurrence of thermal drifts in the electronic offsets and slight variations in the water vapour pressure inside the optical cavity that are caused by water adsorption or desorption from the cell walls. For observation times of a few hours, the absolute stability was measured to be at the kHz level [67].

The offset frequency, *f*, is provided by a RF synthesizer, which in turn is phase locked to the Rb oscillator. The beat-note between reference and probe lasers is first detected by means of a fast photodiode, properly amplified, scaled in frequency, and subsequently compared with the RF signal by means of a digital phase and frequency detector with an integrated loop filter, thus producing an error signal that drives a double-servo system. This latter acts on the diode injection current and on the extended-cavity length of the probe ECDL. Therefore, continuous detection of the beat-note, along with the continuous action of the locking loop, forces the probe laser to maintain a precise frequency-offset from the reference laser, whose maximum value is determined by the bandwidth of the fast photodiode (12 GHz). A step-by-step frequency scan of the probe laser is produced by varying at a constant rate the frequency of the RF source. Typically, the scan is 3100 MHz wide, with a frequency step of 1 MHz and a step-by-step acquisition time of 100 ms.

**Figure 5.**
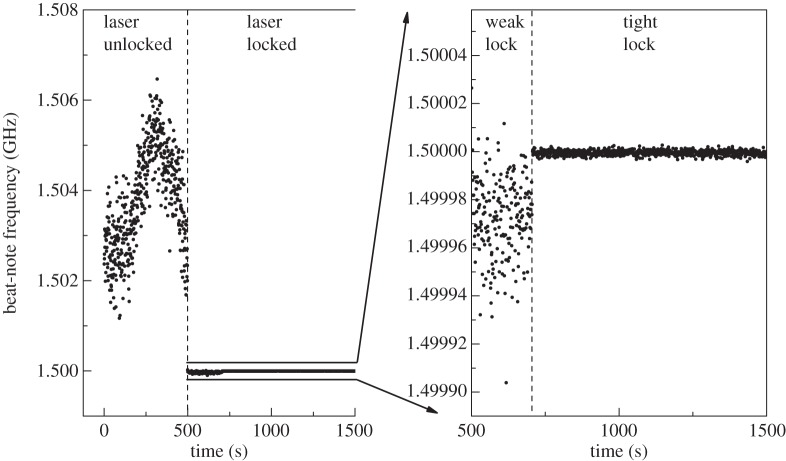
Temporal behaviour of the beat-note frequency, under free-running, weak lock and tight lock conditions of the probe ECDL.

The stability of the probe laser, under the action of the offset-frequency locking loop, is shown in figure 5. Here, the beat-note between probe and reference lasers is continuously monitored by measuring its frequency using a universal counter, with a gate time of 1 s. For the first 500 s, the probe laser is operating under free-running conditions. Then, a weak lock is activated, controlling only the extended-cavity length; finally, a tight lock is performed, thus improving the stability by a factor of 15, when compared with the weak lock. The resulting root-mean-square fluctuation of the beat-note frequency amounts to approximately 1 kHz. An Allan deviation analysis of the dataset of figure 5 provides an indication of the occurrence of white-type frequency noise, under tight lock conditions [49].

In the adopted scheme of frequency stabilization and control, the frequency of the probe ECDL can be written as
6.1
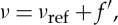
*f*′ being the offset frequency determined by the locking loop. In a variety of operation conditions, it was experimentally verified that *f*′=*f* within the measurement uncertainty of the beat-note frequency, this latter uncertainty being smaller than one part in 10^6^.

**Figure 6.**
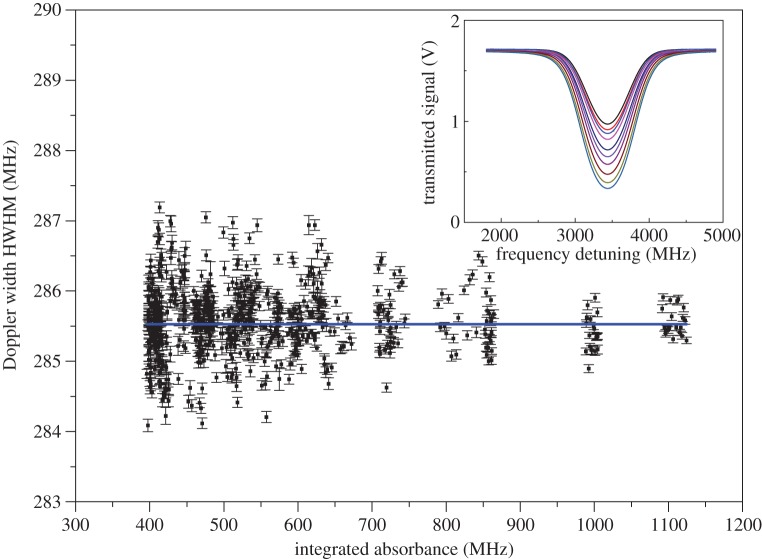
Doppler width determinations from 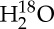 spectra (718, in total). Error bars, corresponding to 1 s.d., are the internal errors resulting from the fitting procedure. Examples of experimental profiles are shown in the inset and refer to a pressure interval between 150 and 500 Pa. The signal-to-noise ratio varied around the value of 5000. A 97.7% enriched ^18^O water sample was used. The gas temperature was 273.1550(3) K, as measured by means of capsule-type standard platinum resistance thermometers. (Online version in colour.)

Nonlinear least-squares fits of experimental spectra, similar to those of the inset of figure 6, using the pcSDHCP, yielded the Doppler width determinations that are shown in figure 6. The observed line shape corresponds to the 4_4,1_ → 4_4,0_ line of the 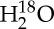 *ν*_1_+*ν*_3_ band at 7199.103190 cm^−1^. This is an ortho transition because it involves quantum states with a total nuclear spin, *I*, equal to 1. Furthermore, it is roughly 3.5 GHz away from the reference transition, this latter being of the para type (namely *I*=0 for the two states). In this case, line-mixing effects can be neglected, because radiative and collisional transitions between ortho and para states are strongly forbidden. Similarly, given the small value of the Einstein coefficient for the spontaneous emission (of about 16 s^−1^ for both lines, namely several orders of magnitude smaller than that typical of electric dipole atomic transitions in the NIR), quantum interference effects can be neglected, contrary to what is observed in precision spectroscopy of helium for the fine structure constant determination [68].

Free parameters in individual fits were the integrated absorbance (*A*), the baseline parameters (*P*_0_ and *P*_1_), the collisional width and the collision frequency (*Γ*_av_ and *β*_av_), both averaged over the absorbers’ speed, the pressure shift (Δ) and the Doppler width (Δ*ν*_D_). The remaining parameters, *η* and *q*, were fixed at 0.2 and 5.017, respectively. In setting the *η* value, the outcomes of [26] were exploited, whereas the choice of the *q* value required a specific study, as extensively explained elsewhere [69]. Very briefly, the *q* exponent was fixed at the value that made the retrieved Doppler width independent from the gas pressure. Taking into account the uncertainty in the slope that derives from a linear fit of the Doppler width data as a function of the integrated absorbance, it turns out that the *q* values yielding a zero slope are those between 5 and 5.034, thus leading to one of the main components of the uncertainty budget for the Boltzmann constant determination that amounts to approximately 15 ppm. Other major sources of uncertainty are the statistical uncertainty (approx. 16 ppm) and the uncertainty associated with the spectral purity of the probe laser (approx. 10 ppm). The complete uncertainty budget has been widely discussed in [69] and recently revised in [70]. The dataset of figure 6 leads to the following value for the Boltzmann constant [12]:

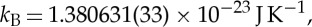
which was derived from the weighted mean of the Doppler widths, the uncertainty being given by the standard deviation of the weighted mean. This value is in full agreement with the recommended Committee on Data for Science and Technology (CODATA) value [71], while being the best determination ever performed by means of an optical method. It is worth noting that the fluctuation in the retrieved Doppler widths is not due to the experimental reproducibility in the spectral acquisitions. By calculating the covariance matrix, it turns out that the Doppler width shows a significant statistical correlation with *Γ*_av_ and *β*_av_. More particularly, the correlation with the collision frequency is responsible for the increase of the statistical uncertainty when moving from the SDVP to the pcSDHCP. The influence of the statistical correlation between Δ*ν*_D_ and *β*_av_ was demonstrated by the results of an interesting test that was done on the acquired spectra, as described hereafter. The spectral analysis was repeated by using the same model (pcSDHCP) and the same code, with the only difference of setting the parameter *β*_av_ to a fixed value, namely the mean value retrieved from the previous fits. The dispersion of the Doppler widths resulted to be smaller by a factor of approximately 2, when compared with figure 6. A similar decrease of the statistical dispersion could be observed by fixing other parameters, such as *Γ*_*av*_. This test confirms the high quality of the recorded experimental profiles. Nevertheless, a further increase of the signal-to-noise ratio in the spectral acquisitions would be necessary to the end of making statistical correlations less effective, even though this requirement could not be sufficient to lower the global uncertainty down to the ppm level.

In order to reduce the statistical uncertainty, but also to minimize the uncertainty associated with the line shape model, a global fitting approach can be of great help [27,72]. A similar approach would allow one to simultaneously fit a manifold of spectra across a given range of pressures, sharing a restricted number of unknown parameters, including the Doppler width, the VC collision frequency per unit pressure, the quantities *m* and *n* characterizing the speed-dependence of collisional broadening and shifting. Exploiting a few relevant physical constraints, which can be easily implemented, the global approach is expected to reduce the statistical correlation among the free parameters, when compared with individual fits. This is, indeed, the case, as demonstrated in a recent study performed on numerically simulated spectra [73]. To this end, a very realistic and sophisticated model, known as the pcSDKS profile [74,75], was used to reproduce NIR 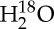 spectra, exactly simulating the situation of the real experiment [12], with the only exception of the signal-to-noise ratio, which was enhanced by nearly a factor of 2. The application of the global fitting approach, based upon the SDHCP, demonstrates that the Doppler width can be determined from 1000 (simulated) spectra with relative precision and accuracy respectively of 0.42 and 0.75 ppm [73].

## 7. Refractive index effects

The Doppler effect of light waves from a moving source is very familiar to physicists. In a gas at thermodynamic equilibrium, because of the molecular chaos, atomic or molecular constituents have an external motion (namely the centre-of-mass motion), which can be treated classically. Instead, the particles have quantized internal degrees of freedom. If one of them is emitting electromagnetic radiation, following an excitation process that removed the particle from its ground state, the wavelength is seen by an observer as blue- or red-shifted depending on whether the particle is moving towards the observer or away. Similarly, if the gaseous sample is invested by electromagnetic radiation (propagating along the *x*-axis), then the angular frequency (*ω*′) that is seen by a moving particle is shifted, with respect to the laboratory frame, according to the equation
7.1
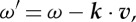
the wavevector being given by 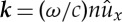, where *n* is the refractive index of the gas. Because light absorption takes place when *ω*′=2*πν*_0_, the Maxwell–Boltzmann distribution of the velocities leads to a Gaussian distribution of light frequencies that are absorbed by the particles. This is the explanation of the well-known Doppler broadening of an absorption line in a gaseous medium. Because, in a rarefied gas, the refractive index is very close to 1, the dependence on *n* is usually neglected, so that the Doppler width is given by equation (1.1). Nevertheless, when pushing the experimental precision to extreme levels, it might be possible to detect a refractive index effect. In reason of that, temperature retrieval should be performed according to the following equation:
7.2
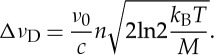
It is useful to remember that such a dependence on the refractive index has recently led to the first observation of the inverse Doppler effect in a negative-index material [76].

In a polar and diamagnetic gas, the refractive index is equal to the square root of the relative dielectric constant of the gas, which in turn is given by the following equation [77]:
7.3
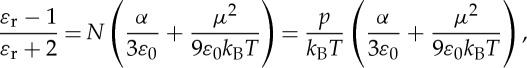
*α* being the molecular polarizability and *μ* the permanent dipole moment. *ε*_r_ is approximately 1 for any rarefied gas; consequently, equation (7.3) reduces to
7.4
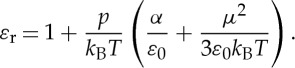
To quantify this effect, one should consider that, for a water vapour sample at a pressure of 300 Pa and a temperature of 273.16 K, the refractive index differs by 1 of about 17 parts over 10^6^ [77]. It should be noted the nonlinear dependence on the gas pressure for such a correction to the Doppler width, differently from the Dicke-narrowing effect that goes linearly with the pressure, at a fixed temperature. Nevertheless, if the pressure range is sufficiently narrow, then a linear approximation holds at the first order, so that the following expression is valid:
7.5
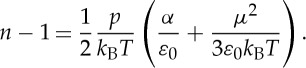
Consequently, it is not possible to distinguish among the two effects, when individual fits are performed. In other words, the refractive index effect acts like a correction to the VC collision frequency. This was the case of the water experiment at 1.39 μm, in which the 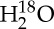 pressure varied between 150 and 500 Pa. In fact, the relative deviation from linearity of *n*−1 versus *p* results to be at the ppm level across the pressure range of interest.

It is worth noting that, in a global fitting approach, the refractive index effect can be properly and accurately considered by using equation (7.4), circumstance that represents a further reason to adopt the global approach. Because, in most cases, the gas pressure is unknown, the correction should we written in terms of integrated absorbance, *A*, as follows:
7.6
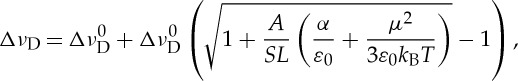

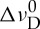
 being the zero-pressure limit of the Doppler width, given by equation (1.1).

At a fixed temperature, if the relative dielectric constant is known (*ε*^0^_r_) for a given reference pressure, for which the integrated absorbance is *A*_0_, equation (7.6) becomes
7.7
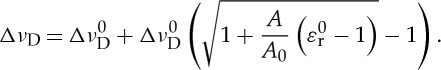
This latter expression can be advantageously used in a global fitting approach, *A* being treated as a free parameter that is characteristic of individual spectra. Obviously, the implementation of equation (7.7) requires the knowledge of the line strength factor at the operation temperature, *T*, and of the absorption path length, in order to determine *A*_0_. Nevertheless, this is not an issue, because *S*(*T*) can be taken from the HITRAN database, for a large variety of molecules [44], whereas *L* can be easily and accurately measured [78]. However, the uncertainty associated with *S*(*T*) (typically varying between 1% and 10%) should enter into the complete uncertainty budget. As a last remark, taking into account the dispersion, namely the dependence of the refractive index on the wavelength, would lead to a second-order correction that must be considered once the target uncertainty of a DBT experiment is ambitiously set below the ppm level.

## 8. Conclusion

This paper has outlined the present status of DBT. It deals with a relatively new method of primary gas thermometry linking the thermal energy to an absolute frequency, namely the line centre frequency of an atomic or molecular resonance, and a frequency interval, which is the Doppler width of the line. Compared with other optical methods, such as total radiation thermometry, it has the advantage of requiring relative intensity measurements of the electromagnetic radiation.

It has been shown how the method could fully exploit the mature technology of laser frequency control and measurement, either in the near- or in the mid-infrared regions. In fact, the DBT methodology has recently benefited from the extremely small uncertainties in building an absolute frequency scale underneath an absorption spectrum. In this respect, two main approaches have been described: one of the them is based upon the technology of optical frequency comb synthesizers, the other one makes use of a reference oscillator that is stabilized against a Doppler-free molecular absorption line. Such a reference oscillator can be based upon an ECDL, a CO_2_ laser or a QCL, as recently demonstrated [79].

As for the thermometric substance, it is surely a matter of discussion whether it is more advantageous to work with atoms or molecules. A low-pressure atomic vapour system, such as Rb or Cs, offers the advantage of the simple structure of its absorption spectrum, when compared with polyatomic molecules. Furthermore, collisional perturbations of the absorption profile can be neglected, because the vapour pressure can be extremely small (approx. 10^−4^ Pa) [80,81]. On the other hand, alkali or alkaline-earth metal atoms present other difficulties, including the strong sensitivity to magnetic fields, the relatively large hyperfine structure and the occurrence of optical pumping effects, which should be carefully accounted for, when modelling the absorption line shape. As a matter of fact, most of the DBT experiments, performed more or less successfully in the last decade, have probed an isolated line of a molecular species. In this respect, advantages and limitations of the main experiments have been highlighted, also making a thoughtful comparison among the different molecules. As a result, it seems clear that non-polar molecules, possibly having strong absorption features in the NIR, should be preferred for many reasons: collisions are less effective; the refractive index is smaller; molecules’ adsorption and desorption from the cell walls are strongly reduced. This latter feature, in particular, makes it possible to perform spectral averaging over long times, thus increasing the signal-to-noise ratio without the risk of line distortion caused by a pressure variation arising from the interaction with the cell.

There exists a line shape problem due to the fact that fully *ab initio* line shape calculations are prohibitively complex for self-colliding molecules. Approximations and simplifications are necessary for the construction of those models that have been mentioned in this article. In the kinetic theory of spectral profiles, in addition to the standard binary collision and impact approximations, there is the assumption that the translation motion can be treated classically [82]. Furthermore, the active molecules (interacting with the electromagnetic field, which is treated classically) are diluted in a bath of structureless perturbers [82]. Therefore, even though a given line shape model is successful in reproducing a high-quality experimental spectrum, the interpretation of some parameters can be problematic, mainly because of statistical correlations among multiple fitted parameters. This is the case, for instance, of Dicke narrowing and speed-dependent broadening parameters in SDGP and SDHCP models. Fortunately, this issue can be significantly reduced by using the global fitting approach for a manifold of spectra, across a given pressure range. This strategy offers the further advantage of providing strong elements for the selection of the most appropriate line shape model, also making DBT more robust, reliable and self-consistent, because it does not require any preliminary knowledge about collisional parameters.

In conclusion, the perspective of approaching the uncertainty level of the most accurate method of primary gas thermometry seems to be real, provided that the effective operation of sophisticated laser-based spectrometers is accompanied by the use of the most refined tools for the nonlinear least-squares analysis of multiple spectra.
